# Supplementary material for: Content-rich biological network constructed by mining PubMed abstracts
Source: BMC Bioinformatics. 2004 Oct 8;5:147. doi: 10.1186/1471-2105-5-147 (PMC528731; doi:10.1186/1471-2105-5-147)
Supplement: Additional File 2 — The original results of the above study (non-essential files are deleted to keep the file size under the limit set by BMC bioinformatics). [file 1471-2105-5-147-S2.bz2 › chilibotAdditionalFile2/dip20/567ID11121055E10931/html/FGF10_FGFR2.html]

 


 **FGF10** and **FGFR2** 
  
Found 47 abstracts in PubMed, retrieved 20.  
 

 What does Google say? 
 PDF only 
| .edu only 

---

**Interactive relationship** (e.g. stimulation, inhibition, etc)

**Neutral relationship**- FGF7 and  **FGF10**  activate only the b isoform of  **FGFR2**  FGFR2b.  Ref: 12591959 Proc Natl Acad Sci U S A, 2003
- These findings suggest that FGF 7 and FGF 10  [ **FGF10** ]  contribute to the regeneration and differentiation of acinar cells and angiogenesis in AP through KGFR  [ **FGFR2** ] .  Ref: 12565793 Exp Mol Pathol, 2002
- FGF1, FGF7, FGF9,  **FGF10** , FGF11, and FGF18 were also expressed in the pancreas at varying time points and levels, as well as FGF receptor  [ **FGFR2** ]  forms FGFR1b, FGFR1c, FGFR2b, FGFR2c, FGFR3b, and FGFR4.  Ref: 12666204 Dev Dyn, 2003
- Keratinocyte growth factor receptor  [ **FGFR2** ]  KGFR  [ **FGFR2** ] , also known as fibroblast growth factor receptor FGFR 2  [ **FGFR2** ]  IIIb, is located in many types of epithelial cells and is activated by four known ligands FGF 1, FGF 3, FGF 7 also known as KGF and FGF 10  [ **FGF10** ]  that are predominantly synthesized by mesenchymal cells.  Ref: 12608893 Pathol Int, 2003

**Non-interactive relationship** (e.g. studied together, co-existance, homology, etc.)

- Fgf7 and  **Fgf10** , the main ligands for  **FgfR2**  IIIb, are expressed in the mesenchyme surrounding the thymic epithelial primordium, and  **Fgf10**  deficient mice also exhibit impaired thymic growth.  Ref: 11489975 J Immunol, 2001
- **FGFR2**  iiib mRNAs were expressed in these cells and the recombinant FGF 10  [ **FGF10** ]  1 ng ml increased the growth rate of COLO 205 cells.  Ref: 11598623 J Nippon Med Sch, 2001
- Our results, in comparison with functional studies in other systems, suggest that FGF 2 FGFR 1, FGF 8 FGFR 2  [ **FGFR2** ]  IIIc and FGF 10  [ **FGF10** ]  FGFR 2  [ **FGFR2** ]  IIIb signaling have different paracrine and juxtacrine functions during SMG initial bud formation and branching.  Ref: 11731698 Cells Tissues Organs, 2002
- In the Twist forelimb bud,  **Fgf10**  expression is reduced, Fgf4 is not expressed, and the domain of Fgf8 and  **Fgfr2**  expression is altered.  Ref: 12142027 Dev Biol, 2002
- Recently it was shown that  **Fgf10** , a major ligand for the IIIb isoform of fibroblast growth factor receptor 2  [ **FGFR2** ]  Fgfr2b, has an important regulatory role in early pancreas development.  Ref: 12559489 Mech Dev, 2003
- In early limb buds, down regulation of Fgf receptor  [ **FGFR2** ]  1 and  **Fgf10**  expression in the mesenchyme occurs concurrent with loss of Fgf4 and Fgf8 expression in the AER.  Ref: 12175489 Mech Dev, 2002
- These findings indicate that FGF 10  [ **FGF10** ]  and its receptor,  **FGFR2**  iiib expression in colorectal adenocarcinoma cells and FGF 10  [ **FGF10** ]  may contribute to the growth of cells of this type.  Ref: 11598623 J Nippon Med Sch, 2001
- **FGF10**  and FGF receptor  [ **FGFR2** ]  FGFR 2  [ **FGFR2** ]  immunostaining showed a similar epithelial expression pattern.  Ref: 12697038 J Endocrinol, 2003
- In later development, FGF3 or  **FGF10**  acting on FGF receptor  [ **FGFR2** ]  2b is likely to be involved in development of the walls of the cochlear spaces, while FGF receptor  [ **FGFR2** ]  3 is involved in differentiation of the pillar cells of the organ of Corti.  Ref: 11914524 Audiol Neurootol< MedlineTA>Audiol Neurootol, 2003
- In the present study, the expression of FGF 10  [ **FGF10** ]  and  **FGFR2**  iiib in five cultured human colorectal adenocarcinoma cell lines COLO 205, DLD 1, HCT 15, SW 480 and WiDr and the localization of FGF 10  [ **FGF10** ]  messenger RNA mRNA and its protein in human colorectal cancer tissues from 10 patients were determined.  Ref: 11598623 J Nippon Med Sch, 2001
